# Association of fascin-1 with mortality, disease progression and metastasis in carcinomas: a systematic review and meta-analysis

**DOI:** 10.1186/1741-7015-11-52

**Published:** 2013-02-26

**Authors:** Vanessa Y Tan, Sarah J Lewis, Josephine C Adams, Richard M Martin

**Affiliations:** 1 School of Social and Community Medicine, University of Bristol, 39 Whatley Road, Bristol BS8 2PS, UK; 2 School of Biochemistry, University of Bristol, Medical Sciences Building, University Walk, Bristol BS8 1TD, UK

**Keywords:** Fascin-1, carcinoma, mortality, metastasis, meta-analysis

## Abstract

**Background:**

Fascin-1 is an actin-bundling protein expressed in many human carcinomas, although absent from most normal epithelia. Fascin-1 promotes filopodia formation, migration and invasion in carcinoma cells; in mouse xenograft tumor models it contributes to metastasis. Fascin-1 is an interesting candidate biomarker for aggressive, metastatic carcinomas but data from individual studies of human tumors have not yet been pooled systematically.

**Methods:**

This systematic review was conducted in accordance with PRISMA guidelines, using fixed and random effects models, as appropriate, to undertake meta-analysis.

**Results:**

A total of 26 immunohistochemical studies of 5 prevalent human carcinomas were identified for meta-analysis. Fascin-1 was associated with increased risk of mortality for breast (pooled hazard ratio, (HR) = 2.58; 95% confidence interval (CI) 1.48 to 4.52; *P *= 0.001), colorectal (HR = 1.60 (1.37 to 1.86; *P *<0.001) and esophageal carcinomas (HR = 1.35; CI 1.13 to 1.60; *P *= 0.001). There was no evidence of association of fascin-1 with mortality in gastric and lung carcinomas. Fascin-1 was associated with increased risk of disease progression in breast (HR = 2.48; CI 1.38 to 4.46; *P *= 0.002) and colorectal carcinomas (HR = 2.12; CI 1.00 to 4.47; *P *= 0.05), but not with progression of lung carcinomas (HR = 0.95; CI 0.49 to 1.85; *P *= 0.9). Fascin-1 was associated with increased risk of lymph node metastasis in colorectal (pooled risk ratio (RR) = 1.47; CI 1.26 to 1.71; *P *<0.001) and gastric carcinomas (RR = 1.43; CI 1.21 to 1.70; *P *<0.001). There was no evidence of association of fascin-1 with lymph node metastasis in lung or esophageal carcinomas. Fascin-1 was associated with increased risk of distant metastasis in colorectal (RR = 1.70; CI 1.18 to 2.45; *P *= 0.004) and gastric carcinomas (RR = 1.93; CI 1.21 to 3.33; *P *= 0.02). No association with distant metastasis in esophageal carcinomas was observed. Pooling across all the carcinomas provided strong evidence for association of fascin-1 with increased risk of mortality (HR = 1.44; CI 1.24 to 1.68; *P *<0.001; n = 3,645), lymph node metastasis (RR = 1.36; CI 1.18 to 1.55; *P *<0.001; n = 2,906) and distant metastasis (1.76; 1.34 to 2.32; *P *<0.001; n = 1,514).

**Conclusions:**

Fascin-1 is associated consistently with increased risk of mortality in breast, colorectal and esophageal carcinomas and with metastasis in colorectal and gastric carcinomas. The results were stable to various sensitivity analyses and did not vary by predefined subgroups. These data will assist rational decision making for focusing investigations of fascin-1 as a biomarker or therapeutic target onto the most relevant carcinomas.

## Background

Biomarkers have a pivotal role in cancer screening, diagnosis, prognosis and therapeutic monitoring. Since tumor metastasis remains a major cause of cancer mortality, there is a compelling need for the discovery and validation of novel biomarkers for early characterization of carcinomas by their aggressive potential [1].

Over the last 10 years, *in vitro*, *in vivo *and clinical immunohistochemical studies have implicated fascin-1 as a novel candidate biomarker for aggressive carcinomas of the biliary duct, bladder, brain, breast, colorectum, endometrium, kidney, liver, lung, neck, esophagus, pharynx, ovary, pancreas, prostate and stomach [2-4]. Fascin-1 is a 55-kDa, actin-bundling protein that plays a key role in the assembly and stability of cell protrusions and other actin-based structures that aid in cell motility, migration and invasion [2,4]. In normal epithelia, fascin-1 is usually absent or present at low levels, yet its expression is increased in colorectal adenomas, esophageal dysplasia and in many carcinomas [2-8]. Fascin-1 increases the migratory capacity of carcinoma cells in culture [9-12]; this is associated with increased invasive and metastatic potential in mouse tumor xenograft models [10-13]. These effects are considered to underlie the observed correlation between fascin-1 expression and clinical aggressiveness in human carcinomas [2-4]. Fascin-1 was recently implicated as the binding partner of the metastasis inhibitory small molecule, macroketone, and this has further increased interest in its potential as a therapeutic target [13,14].

Given the temporal and financial commitments needed to translate basic research to the clinic, it is important to evaluate candidate biomarkers or targets thoroughly at an early stage. Several studies [13,15-17] have investigated the clinical relevance of fascin-1 mRNA levels in tumor tissues. For breast carcinomas, it was reported that high fascin-1 mRNA levels were associated with a lung metastasis signature [16] and decreased overall and metastasis- free survival [13]. However, tissue mRNA is not well-suited to examine the expression of fascin-1 in tumors, because the extracted mRNAs derive from a mixture of cells including myofibroblasts, endothelial cells and dendritic cells that express fascin-1 constitutively [18]. Many immunohistochemical studies of fascin-1 protein in carcinomas have been published. The majority of these immunohistochemical studies have correlated fascin-1 protein in the primary tumor with poor prognosis; however, it is not yet clear if fascin-1 has independent value as a biomarker as the individual studies are not always consistent. For example, immunohistochemical studies of breast carcinomas have reached discrepant conclusions on its association with mortality and metastasis [19-22]. Immunohistochemistry is a complex metric for meta-analysis, due to the use of different scoring systems to assess the extent of fascin-1 staining in tumor specimens, yet studies of fascin-1 have the advantage that almost all publications to date have used the same two antibodies to fascin-1.

To evaluate the hypothesis that fascin-1 can serve as an early marker for identification of the most aggressive carcinomas with metastatic potential, we have conducted a systematic review and meta-analysis of studies that have investigated the clinical implication of fascin-1 in carcinoma progression and patient mortality by immunohistochemistry.

## Methods

### Inclusion and exclusion criteria

A systematic review of all published literature on the association of fascin-1 protein expression with carcinoma progression in breast, colorectal, gastric, lung and esophageal carcinomas was carried out. We focused on these carcinomas because they are the most prevalent carcinomas that are major sources of morbidity and mortality worldwide [23].

Studies had to meet the following inclusion criteria: (1), either randomized controlled trials, cohort or case-control studies; (2), in humans with carcinomas of the breast, colorectum, stomach, lung or esophagus; (3), detected fascin-1 by immunohistochemistry and compared high versus low fascin-1 staining; negative versus positive fascin-1 staining; or negative, low and high fascin-1 staining, and, (4), reported data for at least one of the following outcomes: lymph-node or distal metastasis, disease progression or mortality. We excluded studies investigating tissue mRNA because the extracted mRNAs derive from a mixture of cells including myofibroblasts, endothelial cells and dendritic cells that express fascin-1 constitutively [18]. We excluded case reports, animal and *in vitro *studies.

### Data sources

We searched the Medline, Embase, Web of Science and PubMed bibliographic databases from their inception until April 2012, using a combined text word and MeSH heading search strategy (see Additional file 1 for search terms). We also searched the reference lists of relevant articles and reviews [2-4]. We did not contact any organizations to obtain unpublished results and we did not identify any on-going studies from our systematic review. Authors of conference abstracts were contacted for the published report. We did not apply any language restrictions.

### Selection of studies

The title and abstracts of all retrieved papers were then assessed using the pre-specified inclusion criteria by one author (VYT). Where abstracts were not available or when eligibility was unclear based on the abstract, the full papers were obtained and assessed. Some studies were excluded on the basis of the title or abstract; for all others, full papers were obtained and reviewed by two independent assessors (VYT, RMM), with each assessor blind to the decision of the other. Discrepancies in assigning studies as eligible were resolved by mutual agreement and discussion with JCA. We identified duplicate publications by reviewing study name, authors, location, study population, dates and study design. For multiple study publications from the same patient cohort reporting on similar outcomes, we chose the study with the largest number of cases. For studies that presented different outcomes, we extracted outcomes from both publications.

### Data extraction

Data were extracted onto standardized *pro forma *by one assessor (VYT) and then double-checked by two others (RMM and SJL). Data were extracted on year of publication, study size, mean or median age of patients, location and study design. Different semi-quantitative parameters were used to categorize and dichotomize fascin-1 staining (see Additional file 1 for alternative categorization methods used to assess immunohistochemical staining of fascin-1). Data were extracted on fascin-1 scoring and categorization, the total number of patients in each fascin-1 category and total number of patients experiencing each outcome by fascin-1 category.

For dichotomous outcomes (that is, lymph node metastasis and distant metastasis), the number of patients with the event of interest and total number of patients in each category group (that is, high, low, positive or negative expression) were extracted and the data used to compute risk ratios. If separate data for immunoreactivity or intensity scores were given, we chose to extract the immunoreactivity scores. This is because a recent study [24] showed that immunoreactivity scores alone are a sufficient measure for estimating the association of fascin-1 with mortality. For time to event outcomes (that is, mortality and time-to-disease progression), a hazard ratio was extracted from the study report where possible, otherwise a hazard ratio was estimated from Kaplan Meier curves using the method of Tierney *et al. *[25] (described in Additional file 1). For data extracted from Kaplan-Meier curves, we attempted to check the result with the authors, as there is a potential to overestimate the true number of events [26]. For studies that did not provide a clear definition of their outcomes for time-to-disease progression analysis, we assumed that disease progression was calculated from the date of surgery to the date of disease progression (which is a binary outcome indicating occurrence of mortality, recurrence or metastasis). For time-to-disease progression analysis, a positive hazard ratio (HR) implies an increased risk of disease progression. When a measure of effect (for example, hazard ratio) was adjusted for covariates, it was also extracted along with details of the corresponding covariates.

Where studies included results for each of normal tissue, precursor lesions and carcinomas, we extracted data on fascin-1 in the carcinomas only. For papers or conference abstracts where data were missing or not clear, authors were contacted requesting further information that would enable us to include their data in our meta-analysis. Any disagreements on values of data items extracted were resolved by discussion among VYT, RMM and JCA, or by attempting to contact the authors of the papers for clarification.

### Quality of studies

The methodological quality of studies which presented mortality data was judged and agreed to by three assessors (RMM, SJL and VYT) using the Newcastle-Ottawa scale [27], which was developed to assess the quality of non-randomized cohort studies in meta-analysis. The studies are judged on three broad perspectives using a nine-point scale: the selection of the study group (0 to 4 points), comparability of cohorts (0 to 2 points) and ascertainment of outcome (0 to 3 points).

### Statistical analysis

For our meta-analysis, we stratified the results by method of scoring (that is, low versus high or positive versus negative) and by type of carcinoma. We used the metan command in Stata (StataCorp. 2009. Stata Statistical Software: release 11.2. StataCorp LP; College Station, TX, USA) to calculate a summary hazard ratio (HR) for mortality and time-to-disease progression outcomes or a summary risk ratio (RR) for lymph node and distant metastasis outcomes. We combined fully adjusted effect-estimates if these were available; otherwise, we used the unadjusted estimates.

Heterogeneity was assessed by performing Cochran's Q test. The I^2 ^statistic was calculated as a quantitative measure of the degree of inconsistency across studies, where 0% indicates no heterogeneity and larger percentages indicate increasing heterogeneity [28]. For consistency of presentation throughout our paper, an I^2 ^value of 0 is considered no heterogeneity, an I^2 ^value of 1 to 25% is considered low heterogeneity, an I^2 ^value of 25 to 75% is considered moderate heterogeneity and an I^2 ^value of 75 to 100% is considered as high heterogeneity. We performed fixed-effects meta-analysis unless there was appreciable heterogeneity (I^2 ^value over 50% or chi-squared *P-*value less than 0.1), in which case we also performed a random-effects meta-analysis, which relaxes the assumption of a common treatment effect (that is, effect sizes are assumed to have a normal distribution with variance τ^2^, based on Cochran's Q statistic for heterogeneity).

Subgroup analysis by strata of methodological quality scores (≥6 points, 5 points, <5 points) was carried out to assess whether differences in the quality of the studies were a potential source of heterogeneity for each outcome. Another possible source of heterogeneity was assessed by carrying out subgroup analysis for studies which presented adjusted hazard ratios from multivariable models and for those which presented only unadjusted results.

Sensitivity analysis was carried out for studies which provided a clear definition that mortality referred to death from cancer. For the studies of colorectal carcinomas, sensitivity analysis was carried out for studies which had included an analysis of patients with stage III/IV colorectal carcinomas in their results. Small-study effects were assessed for each outcome by visual inspection of the funnel plot and an Egger's test [29].

## Results

### Identification and selection of included studies

**Figure 1:**
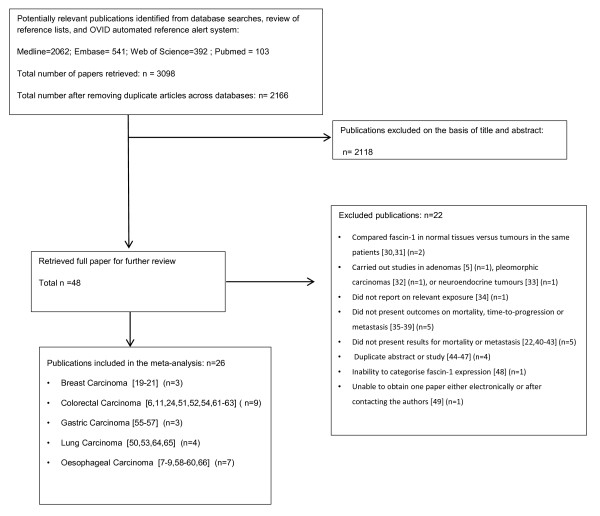
**Flow diagram of the systematic review and meta-analysis process**.

The literature search retrieved a total of 3,098 studies, including 2,062 from MEDLINE, 541 from Embase, 392 from Web of Science and 103 from PubMed. A total of 48 papers were classified initially as potentially relevant and the full papers retrieved. Twenty-two potentially relevant papers were excluded for the following reasons: compared fascin-1 in normal tissues versus tumors in the same patients [30,31]; carried out studies in adenomas [5], pleomorphic carcinomas [32], or neuroendocrine tumors [33]; did not report on relevant exposure [34]; did not present outcomes on mortality, progression or metastasis [35-39]; did not present results for mortality or metastasis [22,40-43]; duplicate abstract or study [44-47], and inability to categorize fascin-1 expression [48]. We were unable to obtain one paper either electronically or after contacting the authors [49]. Overall, 26 papers published between 2003 and 2011 were included in our meta-analysis (Figure 1).

### Characteristics of included studies

Only one study [24] involved a prospectively assembled cohort while the remainder studied retrospectively assembled cohorts. The median sample size of the studies was 128.5 (range 46 to 509) cases, but no studies provided a sample size or power calculation. The characteristics and key results of these studies, as published, are shown in Additional file 2.

### Quality of studies

The methodological quality of the papers was assessed using the Newcastle-Ottawa scaling method [27]. For the 18 studies [6,7,9,19-21,24,50-60] which examined the association of fascin-1 with mortality, 8 studies [6,7,9,24,52,55,56,58] were found to be of high methodological quality (Newcastle-Ottawa scores of above 6 points), 2 studies [50,51] had a score of 5 points which is just below the threshold for high methodological quality and 8 studies [19-21,53,54,57,59,60] were of poor methodological quality (Additional file 1).

For the 6 studies [19,21,50-53] which examined the association of fascin-1 with time-to-disease progression, 1 study [52] was of high methodological quality, 2 studies [50,51] had quality scores just below the 'high' threshold and 3 studies [19,21,53] were of poor methodological quality (Additional file 1).

For the 17 studies [6-9,11,54-58,60-66] that examined the association of fascin-1 with lymph node metastasis, 6 studies [6,7,9,55,56,58] were of high methodological quality and 11 studies [8,11,54,57,60-66] were of poor methodological quality (Additional file 1).

For the 9 studies [8,9,11,52,54,55,57,58,61] which examined the association of fascin-1 with distant metastasis, 4 studies [9,52,55,58] were of high methodological quality and 5 studies [8,11,54,57,61] were of poor methodological quality (Additional file 1).

## Meta-analysis results

### Breast carcinoma

**Figure 2:**
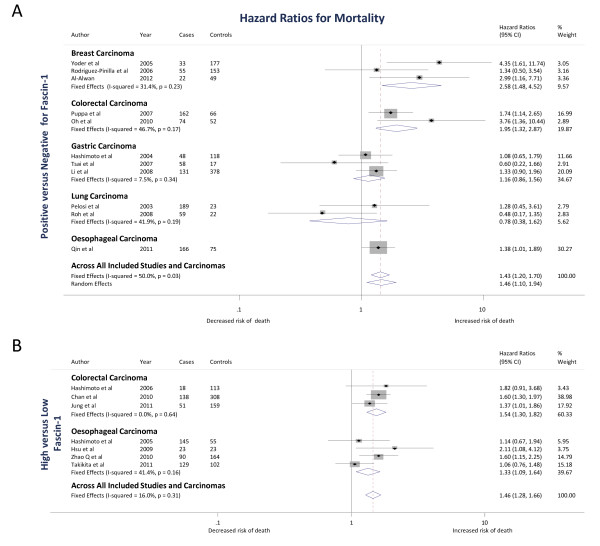
**The association of fascin-1 with mortality in breast, colorectal, gastric, lung and esophageal carcinomas**. **The Forest plots show **(**A**) Positive versus negative = positive fascin-1 staining versus negative fascin-1 staining; (**B**) High versus low = High fascin-1 staining versus low fascin-1 staining (see Methods for details of the scoring categorizations). In both A and B, squares indicate the study-specific effect estimate, with the size proportional to the inverse of the variance (I-V); horizontal lines show study-specific 95% confidence intervals. The diamonds are pooled estimates and their 95% confidence intervals, based on random or fixed effects meta-analysis models. The dashed vertical line is the overall pooled estimate across all included studies and carcinomas.

Three studies [19-21] examined the association of fascin-1 with mortality, with a total of 489 breast carcinoma cases. The pooled fixed effects HR estimate for fascin-1 positivity (that is, scored as positive versus negative) of the carcinomas was 2.58 (1.48 to 4.52; *P *= 0.001) (Figure 2A). There was moderate heterogeneity between the three cohort studies (I^2 ^= 31.4%), although the statistical evidence supporting heterogeneity was weak (*P *= 0.23).

**Figure 3:**
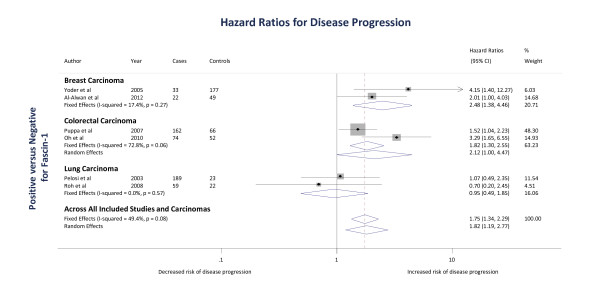
**The association of fascin-1 with disease progression in breast, colorectal and lung carcinomas**. In the Forest plot, positive versus negative = positive fascin-1 staining versus negative fascin-1 staining. Squares indicate the study-specific effect estimate, with the size proportional to the inverse of the variance (I-V); horizontal lines show study-specific 95% confidence intervals. The diamonds are pooled estimates and their 95% confidence intervals, based on random or fixed effects meta-analysis models. The dashed vertical line is the overall pooled estimate across all included studies and carcinomas. Time-to-disease progression was calculated from the date of surgery to the date of disease progression.

Two studies [19,21] examined the association of fascin-1 positivity of carcinomas with time-to-disease progression, with a total of 281 breast carcinoma cases. Based on studies examining fascin-1 positivity, the pooled fixed effects HR estimate was 2.48 (1.38 to 4.46; *P *= 0.002) for mortality, recurrence or metastasis outcomes (Figure 3). There was low heterogeneity between the two cohort studies (I^2 ^= 17.4%; *P *= 0.27).

### Colorectal carcinoma

Two studies [51,52] examined the association of fascin-1 positivity of carcinomas with mortality, and three [6,24,54] examined the association of high fascin-1 with mortality, with a total of 1,141 colorectal carcinoma cases. The study by Ozerhan *et al. *[61] was excluded from the analysis as it had presented two-year mortality rates for each fascin-1 category, and we could not estimate hazard ratios. For studies examining fascin-1 positivity of the carcinomas, the pooled fixed effects HR estimate was 1.95 (1.32 to 2.87; *P *= 0.001) (Figure 2A). Based on studies examining high fascin-1 expression, the pooled fixed effects HR estimate was 1.54 (1.3 to 1.82; *P *<0.001) (Figure 2B). Pooling across all studies gave a pooled fixed effects HR estimate of 1.60 (1.37 to 1.86; *P *<0.001). There was moderate heterogeneity between studies that scored fascin-1 as positive versus negative (I^2 ^= 46.7%; *P *= 0.17). There was no heterogeneity between studies that scored fascin-1 as high vs low levels (I^2 ^= 0%; *P *= 0.64) or when all studies were pooled (I^2 ^= 0.0%; *P *= 0.41).

Two studies [51,52] examined the association of fascin-1 positivity of carcinomas with time-to-disease progression, with a total of 354 colorectal carcinoma cases. Based on studies examining fascin-1 positivity, the pooled random effects HR estimate was 2.12 (1.00 to 4.47; *P *= 0.05) for mortality, recurrence or metastasis outcomes (Figure 3). There was moderate-to-high heterogeneity between the two cohort studies (I^2 ^= 73%; *P *= 0.06).

**Figure 4:**
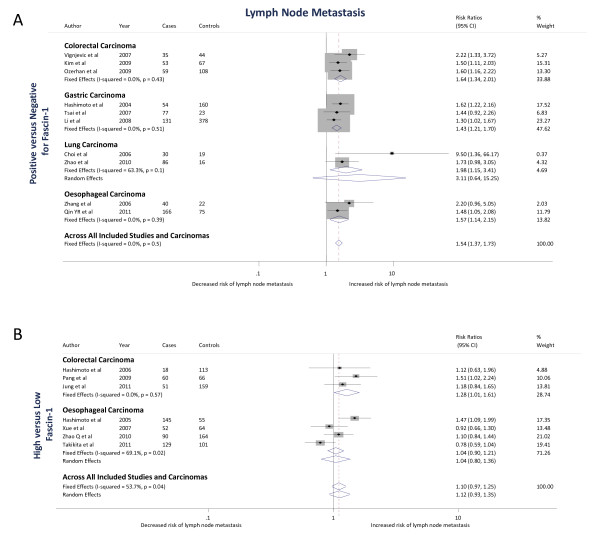
**The association of fascin-1 with lymph node metastasis in colorectal, gastric, lung and esophageal carcinomas**. The Forest plots show (**A**) Positive versus negative = positive fascin-1 staining versus negative fascin-1 staining; (**B**) High versus low = high fascin-1 staining versus low fascin-1 staining. In both A and B, squares indicate the study-specific effect estimate, with the size proportional to the inverse of the variance (I-V); horizontal lines show study-specific 95% confidence intervals. The diamonds are pooled estimates and their 95% confidence intervals, based on random or fixed effects meta-analysis models. The dashed vertical line is the overall pooled estimate across all included studies and carcinomas.

Three studies [11,61,62] examined the association of fascin-1 positivity of colorectal carcinomas with lymph node metastasis and 3 [6,54,63] examined the association of high fascin-1 with lymph node metastasis, with a total of 833 colorectal carcinoma cases. Based on studies examining fascin-1 positivity, the pooled fixed effects RR estimate was 1.64 (1.34 to 2.01; *P *<0.001) (Figure 4A). Based on studies examining high fascin-1 expression, the pooled fixed effects RR estimate was 1.28 (1.01 to 1.61; *P *= 0.04) (Figure 4B). Pooling across all studies gave a pooled fixed effects RR estimate of 1.47 (1.26 to 1.71; *P *<0.001). There was no heterogeneity between studies that scored fascin-1 as positive versus negative (I^2 ^= 0%, *P *= 0.4), high versus low levels (I^2 ^= 0%, *P *= 0.6) or when all studies were pooled (I^2 ^= 6.4%; *P *= 0.38).

**Figure 5:**
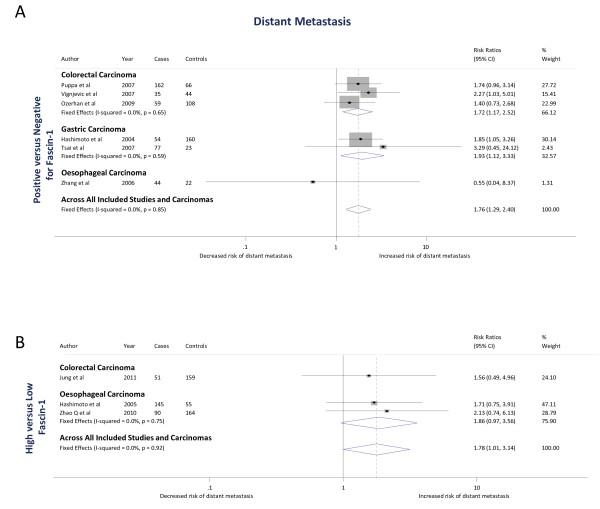
**The association of fascin-1 with distant metastasis in colorectal, gastric and oesophageal carcinomas**. The Forest plots show (**A**) Positive versus negative = positive fascin-1 staining versus negative fascin-1 staining; (**B**) High versus low = high fascin-1 staining versus low fascin-1 staining. In both A and B, squares indicate the study-specific effect estimate, with the size proportional to the inverse of the variance (I-V); horizontal lines show study-specific 95% confidence intervals. The diamonds are pooled estimates and their 95% confidence intervals, based on random or fixed effects meta-analysis models. The dashed vertical line is the overall pooled estimate across all included studies and carcinomas.

Three studies [11,52,61] examined the association of fascin-1 positivity of carcinomas with distant metastasis and one [54] examined the association of high fascin-1 with distant metastasis, with a total of 684 colorectal carcinoma cases. The study by Oh *et al. *[51] was excluded from the meta-analysis as it had presented adjusted HRs and could not be pooled with the RRs estimated in the above studies. Based on studies examining fascin-1 positivity, the pooled fixed effects RR estimate was 1.72 (1.17 to 2.52; *P *= 0.006) (Figure 5A). The study examining high fascin-1 had a RR estimate of 1.56 (0.49 to 4.96; *P *=0.4) (Figure 5B). Pooling across all studies gave a fixed effects RR estimate of 1.70 (1.18 to 2.45; *P *= 0.004). There was no heterogeneity between studies that scored fascin-1 as positive versus negative (I^2 ^= 0%; *P *= 0.65) or when all studies were pooled (I^2 ^= 0%; *P *= 0.83).

### Gastric carcinoma

Three studies [55-57] examined the association of fascin-1 positivity of carcinomas with mortality, with a total of 750 gastric carcinoma cases. Based on the studies, the pooled fixed effects HR estimate was 1.16 (0.86 to 1.56; *P *= 0.33) (Figure 2A). There was low heterogeneity between the three cohort studies (I^2 ^= 8%; *P *= 0.34).

Three studies [55-57] examined the association of fascin-1 positivity of carcinomas with lymph node metastasis, with a total of 823 gastric carcinoma cases. Based on the studies, the pooled fixed effects RR estimate was 1.43 (1.21 to 1.70; *P *<0.001) (Figure 4A). There was no heterogeneity between the three cohort studies (I^2 ^= 0%; *P *= 0.5).

Two studies [55,57] examined the association of fascin-1 positivity of carcinomas with distant metastasis, with a total of 314 gastric carcinoma cases. Based on the studies, the pooled fixed effects RR estimate was 1.93 (1.12 to 3.33; *P *= 0.02) (Figure 5A). There was no heterogeneity between the two cohort studies (I^2 ^= 0%; *P *= 0.6).

### Lung carcinoma

Two studies [50,53] examined the association of fascin-1 positivity of carcinomas with mortality, with a total of 293 lung carcinoma cases. Based on the studies, the pooled fixed effects HR estimate was 0.78 (0.38 to 1.62; *P *= 0.51) (Figure 2A). There was moderate heterogeneity between the two cohort studies (I^2 ^= 42%; *P *= 0.2).

Two studies [50,53] examined the association of fascin-1 positivity of carcinomas with time-to-disease progression with a total of 293 lung carcinoma cases. Based on the studies, the pooled fixed effects HR estimate was 0.95 (0.49 to 1.85; *P *= 0.9) for mortality, recurrence or metastasis outcomes (Figure 3). There was no heterogeneity between the two cohort studies (I^2 ^= 0%; *P *= 0.6).

Two studies [64,65] examined the association of fascin-1 positivity of carcinomas with lymph node metastasis, with a total of 147 lung carcinoma cases. Based on the studies, the pooled random effects RR estimate was 3.11 (0.64 to 15.25; *P *= 0.2) (Figure 4A). There was moderate heterogeneity between the two cohort studies (I^2 ^= 63%; *P *= 0.1).

### Esophageal carcinoma

Four studies [7,9,58,59] examined the association of high fascin-1 with mortality and one [60] examined the association of fascin-1 positivity of carcinomas with mortality, with a total of 972 esophageal carcinoma cases. The study examining fascin-1 positivity had a HR estimate of 1.38 (1.01 to 1.89; *P *= 0.05) (Figure 2A). Based on studies examining high fascin-1, the pooled fixed effects HR estimate was 1.33 (1.09 to 1.64; *P *= 0.01) (Figure 2B). Pooling across all studies gave a fixed effects HR estimate of 1.35 (1.13 to 1.60; *P *= 0.001). There was moderate heterogeneity between studies that scored fascin-1 as high versus low levels (I^2 ^= 41%; *P *= 0.2) and moderate heterogeneity when all studies were pooled (I^2 ^= 22.3%; *P *= 0.27).

Two studies [8,60] examined the association of fascin-1 positivity of carcinomas with lymph node metastasis and four [7,9,58,66] examined the association of high fascin-1 with lymph node metastasis, with a total of 1,103 esophageal carcinoma cases. Based on studies examining fascin-1 positivity, the pooled fixed effects RR estimate was 1.57 (1.14 to 2.15; *P *= 0.01) (Figure 4A). Based on studies examining high fascin-1, the pooled random effects RR estimate was 1.04 (0.80 to 1.36; *P *= 0.77) (Figure 4B). Pooling across all studies gave a random effects RR estimate of 1.16 (0.91 to 1.49; *P *= 0.24). There was no heterogeneity between studies that scored fascin-1 as positive versus negative (I^2 ^= 0%; *P *= 0.4). There was moderate heterogeneity between studies that scored fascin-1 as high versus low levels (I^2 ^= 69.1%; *P *= 0.02) or when all studies were pooled (I^2 ^= 68.1%; *P *= 0.008).

Two studies [9,58] examined the association of high fascin-1 with distant metastasis and one [8] examined the association of fascin-1 positivity of carcinomas with distant metastasis, with a total of 516 esophageal carcinoma cases. The study examining fascin-1 positivity had a RR estimate of 0.55 (0.04 to 8.37; *P *= 0.7) (Figure 5A). Based on studies examining high fascin-1, the pooled fixed effects RR estimate was 1.86 (0.97 to 3.56; *P *= 0.06) (Figure 5B). Pooling across all studies gave a pooled fixed effects RR estimate of 1.74 (0.92 to 3.28; *P *= 0.09). There was no heterogeneity between studies that scored fascin-1 as high versus low levels (I^2 ^= 0%; *P *= 0.8) or when all studies were pooled (I^2 ^= 0%; *P *= 0.66).

### Analysis of all included carcinomas

Eleven studies [19-21,50-53,55-57,60] examined the association of fascin-1 positivity of carcinomas with mortality and seven studies [6,7,9,24,54,58,59] examined the association of high fascin-1 with mortality, with a total of 3,645 carcinoma cases. Based on studies examining fascin-1 positivity, the pooled random effects HR estimate was 1.46 (1.10 to 1.94; *P *= 0.008) (Figure 2A). Based on studies examining high fascin-1, the pooled fixed effects HR estimate was 1.46 (1.28 to 1.66; *P *<0.001) (Figure 2B). The overall pooled random effects HR estimate for the association of fascin-1 with mortality was 1.44 (1.24 to 1.68; *P *<0.001). There was low heterogeneity between studies that scored fascin-1 as high versus low levels (I^2 ^= 16%; *P *= 0.31). There was moderate heterogeneity between studies that scored fascin-1 as positive versus negative (I^2 ^= 50%; *P *= 0.03) or when all studies were pooled (I^2 ^= 37.4%; *P *= 0.06).

Six studies [19,21,50-53] examined the association of fascin-1 positivity with time-to-disease progression, with a total of 928 carcinoma cases. The overall random effects HR estimate for the association of fascin-1 with time-to-disease progression was 1.82 (1.19 to 2.77; *P *= 0.006) for mortality, recurrence or metastasis outcomes (Figure 3). There was moderate heterogeneity between the studies (I^2 ^= 49.4%; *P *= 0.08).

Ten studies [8,11,55-57,60-62,64,65] examined the association of fascin-1 positivity of carcinomas with lymph node metastasis and seven studies [6,7,9,54,58,63,66] examined the association of high fascin-1 with lymph node metastasis, with a total of 2,906 carcinoma cases. Based on studies examining fascin-1 positivity, the pooled fixed effects RR estimate was 1.54 (1.37 to 1.73; *P *<0.001) (Figure 4A). Based on studies examining high fascin-1, the pooled random RR estimate was 1.12 (0.93 to 1.35; *P *= 0.25) (Figure 4B). The overall pooled random effects RR estimate for the association of fascin-1 with lymph node metastasis was 1.36 (1.18 to 1.55; *P *<0.001). There was no heterogeneity between studies that scored fascin-1 as positive versus negative (I^2 ^= 0%; *P *= 0.5). There was moderate heterogeneity between studies that scored fascin-1 as high versus low levels (I^2 ^= 53.7%; *P *= 0.04 or when all studies were pooled (I^2 ^= 55.3%; *P *= 0.003).

Six studies [8,11,52,55,57,61] examined the association of fascin-1 positivity of carcinomas with distant metastasis and three studies [9,54,58] examined the association of high fascin-1 with distant metastasis, with a total of 1,514 carcinoma cases. Based on studies examining fascin-1 positivity, the pooled fixed effects RR estimate was 1.76 (1.29 to 2.4; *P *<0.001) (Figure 5A). Based on studies examining high fascin-1, the pooled fixed RR estimate was 1.78 (1.0 to 3.14; *P *= 0.05) (Figure 5B). The overall pooled fixed effects RR estimate for the association of fascin-1 with distant metastasis was 1.76 (1.34 to 2.32; *P *<0.001). There was no heterogeneity between studies that scored fascin-1 as positive versus negative (I^2 ^= 0%; *P *= 0.85), between studies that scored fascin-1 as high versus low levels (I^2 ^= 0%; *P *= 0.92) or when all studies were pooled (I^2 ^= 0%; *P *= 0.98).

### Subgroup analysis

To address if the variations in methodological quality of the studies were possible sources of heterogeneity, we carried out subgroup analysis by methodological quality scores. For the 18 studies [6,7,9,19-21,24,50-60] which examined the association of fascin-1 with mortality, 8 studies [6,7,9,24,52,55,56,58] with a high quality scores had a pooled fixed effects HR estimate of 1.43(1.26 to 1.63; *P *<0.001) (Table 1A), 2 studies [50,51] with quality scores just below the threshold had a pooled random effects HR estimate of 2.20 (0.77 to 6.34; *P *= 0.14) (Table 1A) and 8 studies [19-21,53,54,57,59,60] with poor quality scores had a random effects HR of 1.48 (1.05 to 2.08; *P *= 0.03) (Table 1A).

**Table 1:** Subgroup analyses

Strata of analysis for each outcome	Number of studies included	HR/RR as appropriate (95% CI); *P*-value	Heterogeneity
**A. Methological quality scores**			
Mortality			
Studies with a score of ≥6 points [6,7,9,24,52,55,56,58]	8	1.43 (1.26 to 1.63); *P *<0.001	I^2 ^= 12.4%; *P *= 0.33
Studies with a score of 5 points [50,51]	2	2.20 (0.77 to 6.34); *P *= 0.14	I^2 ^= 52.6%; *P *= 0.15
Studies with a score of <5 points [19-21,53,54,57,59,60]	8	1.48 (1.05 to 2.08); *P *= 0.03	I^2 ^= 55.5%; *P *= 0.03
			
Time-to-disease progression			
Study with a score of ≥6 points [52]	1	1.52 (1.04 to 2.23); *P *<0.05	
Studies with a score of 5 points [50,51]	2	1.91 (0.64 to 5.70); *P *= 0.25	I^2 ^= 77.4%; *P *= 0.04
Studies with a score of <5 points [19,21,53]	3	1.90 (0.81 to 4.46); *P *= 0.14	I^2 ^= 54.9%; *P *= 0.11
			
Lymph node metatasis			
Study with a score of ≥6 points [6,7,9,55,56,58]	6	1.21 (0.97 to 1.51); *P *= 0.09	I^2 ^= 68.5%; *P *= 0.007
Studies with a score of <5 points [8,11,54,57,60-66]	11	1.47 (1.25 to 1.73); *P *<0.001	I^2 ^= 38.5%; *P *= 0.09
			
Distant metastasis			
Studies with a score of ≥6 points [9,52,55,58]	4	1.81 (1.28 to 2.57); *P *= 0.001	I^2 ^= 0.0%; *P *= 0.99
Studies with a score of <5 points [8,11,54,57,61]	5	1.68 (1.08 to 2.62); *P *= 0.02	I^2 ^= 0.0%; *P *= 0.74
			
**B. Adjusted versus unadjusted results for Mortality**			
Studies with results from multivariable analysis [6,7,9,24,51,52,54-56,58]	10	1.44 (1.28 to 1.62); *P *<0.001	I^2 = ^21.7%; *P *= 0.24
Studies without results from multivariable analysis [19-21,50,53,57,59,60]	8	1.48 (0.97 to 2.26); *P *= 0.07	I^2 = ^55.3%; *P *= 0.03

Note: Time-to-disease progression is defined by the occurrence of the outcomes time-to-mortality, recurrence or metastasis.

For the 6 studies [19,21,50-53] which examined the association of fascin-1 with time-to-disease progression, 1 study [52] with a high quality score had a HR of 1.52 (1.04 to 2.23; *P *<0.05), 2 studies [50,51] with quality scores just below the threshold had a pooled random effects HR estimate of 1.91 (0.64 to 5.70; *P *= 0.25) (Table 1A) and 3 studies [19,21,53] with poor quality scores had a pooled random effects HR estimate of 1.90 (0.81 to 4.46; *P *= 0.14) (Table 1A).

For the 17 studies [6-9,11,54-58,60-66] which examined the association of fascin-1 with lymph node metastasis, 6 studies [6,7,9,55,56,58] with high quality scores had a pooled random effects RR estimate of 1.21 (0.97 to 1.51; *P *= 0.09) (Table 1A) and 11 studies [8,11,54,57,60-66] with poor quality scores had a pooled random effects RR estimate of 1.47 (1.25 to 1.73; *P *<0.001**) (**Table 1A).

For the 9 studies [8,9,11,52,54,55,57,58,61] which examined the association of fascin-1 with distant metastasis, 4 studies [9,52,55,58] with high quality scores had a fixed effects RR estimate of 1.81(1.28 to 2.57; *P *= 0.001) (Table 1A) and 5 studies [8,11,54,57,61] with poor quality scores had a fixed effects RR estimate of 1.68 (1.08 to 2.62; *P *= 0.02) (Table 1A).

In addition, we grouped the studies according to whether they had presented adjusted or unadjusted results. Ten studies [6,7,9,24,51,52,54-56,58] presented multivariable results for the mortality outcome, with a total of 2,501 carcinoma cases. The overall pooled fixed effects HR estimate was 1.44 (1.28 to 1.62; *P *<0.001) (Table 1B). There was low heterogeneity between the studies (I^2 ^= 21.7%; *P *= 0.24). Eight studies [19-21,50,53,57,59,60] presented unadjusted results which included results from univariable analysis and univariable Kaplan-Meier curves. The overall pooled random effects HR estimate was 1.48 (0.97 to 2.26; *P *= 0.07) (Table 1B). There was moderate heterogeneity between the studies (I^2 ^= 55.3%; *P *= 0·03).

### Sensitivity analysis

The analysis of all carcinomas indicated an association of fascin-1 with increased risk of mortality. However, not all studies had provided a clear definition of the cause of death. Therefore, we carried out a sensitivity analysis that included only studies [6,9,19,20,51,52,54,55,58,60] which had provided a clear definition that "death" referred to cancer-specific mortality. The result was consistent with the analysis of all mortality outcomes (pooled fixed effects HR = 1.49 (1.29 to 1.72); *P *<0.001) (Table 2A). A second sensitivity analysis included only studies of patients with stage III/IV colorectal carcinomas [6,24,51,52]. The result (pooled fixed effects HR = 1.70 (1.42 to 2.03); *P *<0.001) was consistent with the analyses of all stages of colorectal carcinomas (Table 2B).

**Table 2:** Sensitivity analyses

Strata of analysis for each outcome	Number of studies included	HR (95% CI); P-value	Heterogeneity
A. Studies with definition for cancer specific mortality included [6,9,19,20,51,52,54,55,58,60]	10	1.49 (1.29 to 1.72); *P *<0.001	I^2^:23.4%; *P *= 0.23
B. Only Stage III/IV colorectal carcinoma studies included [6,24,51,52]	High versus Low: 2	High versus Low: 1.64 (1.34 to 2.00); *P *<0.001	I^2^: 0%; *P *= 0.38
	Positive versus Negative: 2	Positive versus Negative: 1.95 (1.32 to 2.87); *P *= 0.001	I^2^: 46.7%; *P *= 0.17
	Overall colorectal studies: 4	Overall colorectal studies: 1.70 (1.42 to 2.03); *P *<0.001	I^2^: 7.2%; *P *= 0.36

### Publication bias

**Figure 6:**
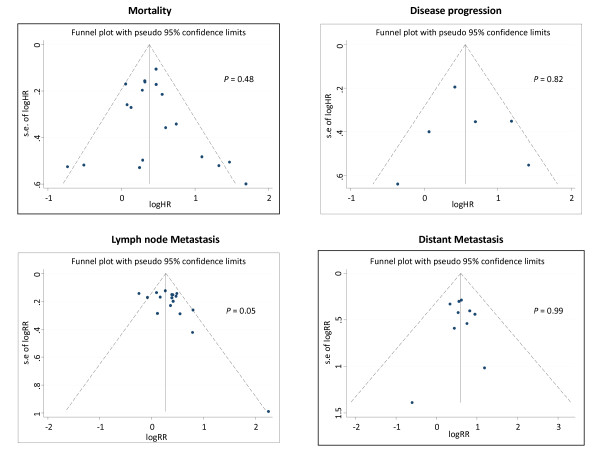
**Assessment of publication bias for mortality, disease progression analysis, lymph node metastasis and distant metastasis**. In each funnel plot analysis, black dots represent each study's effect estimate (drawn on a log scale) plotted against its standard error. The outer dashed lines represent the 95% confidence limits around the summary effect estimate, within which 95% of studies are expected to lie in the absence of either biases or heterogeneity. *P-*values are from the results of Egger's test to assess publication bias.

We investigated for the potential presence of publication bias through visual inspection of funnel plot asymmetry and by computation of Egger's test. For the mortality, time-to-disease progression and distant metastasis outcomes, data points in the funnel plot analyses approximated a symmetrical distribution indicating that publication bias is unlikely to be present in our analyses (Figure 6). For the lymph node metastasis outcome Egger's *P*-value for funnel plot asymmetry was 0.05. Visual inspection of the funnel plot for lymph node metastasis, (Figure 6), showed that two studies, [8,64], were highly influential in the result of Egger's asymmetry test. After exclusion of these two studies, the data points in the funnel plot analysis approximated a symmetrical distribution with no evidence of bias (Egger's test: *P *= 0.27) (Additional file 3).

## Discussion

The importance of demonstrating the reproducibility of biomarker studies to validate their utility for the clinic is increasingly well recognized [67]. Our systematic review and meta-analysis is the first investigation of the overall worldwide evidence on the association of the actin-bundling protein fascin-1 with mortality, time-to-disease progression, lymph node metastasis and distant metastasis, in some of the most prevalent forms of carcinoma.

Our meta-analyses demonstrate that there is strong evidence that fascin-1 protein is associated with an up to two and a half-fold increased risk of mortality in breast, colorectal and esophageal carcinomas. At present, there is little evidence that fascin-1 is associated with mortality for gastric and lung carcinomas. Fascin-1 is correlated with increased risk of disease progression in breast and colorectal carcinomas, but not in lung carcinoma. Strong evidence for association of fascin-1 with increased risk of lymph node metastasis was found for colorectal and gastric carcinomas, but not for lung and esophageal carcinomas. Fascin-1 protein was also associated with a greater than 70% increased risk of distant metastasis in colorectal, gastric and esophageal carcinomas, although the statistical evidence for association with esophageal carcinoma metastasis was weak.

The potential for search and inclusion bias in our meta-analysis is considerably reduced as we conducted a comprehensive search of four databases for papers published in all languages. Publication bias could result from selective reporting of results by individual studies, because statistically insignificant results are often not published [68,69]. Many authors did not respond to requests for information or clarification and we also identified several studies which did not report all their data. However, funnel plot analyses were not generally indicative of any strong publication bias because visual inspection of funnel plots did not show asymmetry (Figure 6). There was potential for misclassification of outcome, because some studies did not provide a clear definition of the cause of death. However, sensitivity analysis of studies that had provided a clear definition of cancer-specific mortality demonstrated that fascin-1 expression was associated with a 49% increased risk of cancer-specific death. This effect-estimate was similar to the pooled results from studies that did not have a clear definition of the cause of death (Table 2A).

All esophageal carcinoma studies included in our meta-analysis were carried out in Asia. Esophageal carcinoma shows marked geographic variation with exceptionally high rates in Asia [70]. However, rates of esophageal adenocarcinomas are increasing rapidly in several Western countries [71]. To exclude ethnicity and locality as modifying factors, more studies should be carried out to assess the association between fascin-1 and risk of esophageal cancer-specific mortality in Western countries.

All the included studies used immunohistochemical analysis of archived tumor specimens to quantify fascin-1. Immunohistochemistry is a pathologist-based scoring system that is the most commonly used and reliable technique in diagnostic histopathology [72]. In addition, nearly all included studies used the same two antibodies to fascin-1, enabling comparability of prognostic effects in our meta-analysis. Only one cohort [24] was prospectively assembled. The remaining studies were retrospective in design using samples based on the availability of specimens with interpretable cores (which may have been non-random) and clinical histories, rather than specifying and recruiting a truly representative sample from a clearly defined target population. No studies provided an appropriate justification of the sample size or a power calculation. In addition, there was limited control for confounding in the analysis of most datasets and incomplete ascertainment of outcome data, due to loss to follow-up, which can lead to biased effect-estimates. Therefore, most of the studies included in our meta-analysis were assessed as having a risk of bias. For the 18 studies examining associations of fascin-1 with mortality, only 8 studies, [6,7,9,24,52,55,56,58], were found to be of high methodological quality. We were somewhat reassured, however, that the high quality studies showed a positive association between fascin-1 expression and mortality (pooled fixed effects HR: 1.43 (1.26 to 1.63; *P *<0.001), that was similar in magnitude to the association observed when all 18 studies were pooled (pooled random effects HR: 1.44 (1.24 to 1.68; *P *<0.001).

The scoring of fascin-1 by immunohistochemistry is a continuous measurement and in most publications researchers categorized tumor specimens into high/positive fascin-1 or low/negative fascin-1 based on different semi-quantitatively assessed cut-off points. For example, two studies [58,66] categorized 0 to 8 as low expression and 9 to 12 as high expression.

On the other hand, another study [9] dichotomized low expression as a score of <75% immunoreactive tumor cells and high expression as >75% immunoreactive tumor cells. These differences could possibly discard potentially important quantitative information and reduce statistical power to detect real associations [73]. There was also qualitative evidence of heterogeneity between the individual studies, but based on the number of available studies for each carcinoma type, it was not possible to investigate this statistically using meta-regression (apart from stratifying by scoring method). However, a recent meta-analysis of other immunohistochemical prognostic markers demonstrated that the cut-off value of the percentage of positively-stained cells used in the scoring criteria appears unlikely to bias the underlying relationship between a prognostic biomarker and mortality [74]. With regard to future studies of fascin-1, our analyses indicate that scoring of tumors as fascin-1 negative (taken either as completely absent [52,61], below 5% fascin-1 positive cells within a tumor [21,50,53,55-57,60,64], or below 10% fascin-1 positive cells within a tumor [19,20,51,62]) versus fascin-1 positive (that is, either more than 5% or 10% fascin-1 positive cells within a tumor) is adequate to detect an association of fascin-1 with risk of mortality (Figure 2). The validity of this simple scoring metric should facilitate clinical application of fascin-1 as a biomarker.

Metastasis is the major source of cancer-related mortality and, at the cellular level, a major effect of fascin-1 is in promoting carcinoma cell migration and invasion. Promotion of metastasis by fascin-1 has been reported in several mouse tumor xenograft models [10-13]. Pooling across all the included carcinomas in our dataset, we obtained strong evidence that fascin-1 is associated with increased risk of mortality, lymph node metastasis and distant metastasis. However, there was evidence of heterogeneity between different carcinomas. This could be expected due to the biological differences in mechanisms of carcinoma progression for different organs. For example, the weak association between fascin-1 and metastasis in esophageal carcinomas could be due to the rapid progression to metastatic disease that occurs in most patients with esophageal carcinoma. This is due to the unique anatomy of the esophagus which lacks serosa, thus making it easier for early rapid tumor cell spread through the extensive network of lymph nodes [75,76]. This disease course is very different from that of breast or colon carcinomas that develop through distinct stages to an aggressive phenotype over many years.

Heterogeneity was also apparent within carcinoma types. The three breast carcinoma studies showed evidence of heterogeneity for mortality (I^2 ^= 31.4%). However, it should be noted that each study included different histological types of breast carcinomas. Yoder *et al. *[19] studied primary node-positive and node-negative invasive breast carcinomas, which included infiltrating ductal carcinomas, infiltrating lobular carcinomas, mucinous carcinomas and medullary carcinomas. Al-Alwan *et al. *[21] studied invasive ductal carcinoma only, and Rodriguez-Pinilla *et al. *[20] investigated node-negative sporadic and hereditary invasive breast carcinomas. As breast cancer is a clinically heterogeneous disease [77], the heterogeneity identified in the meta-analysis could be due to the pooling of results across different subtypes of breast carcinomas. Further analyses of the association of fascin-1 with breast cancer mortality or metastasis will need to specifically evaluate different sub-types of breast carcinomas. In contrast, all the esophageal carcinoma studies had analyzed squamous cell carcinomas (Additional file 2); thus the between study heterogeneity is not due to pooling of results across biologically different tumors within the same organ.

Heterogeneity was also evident between the lung carcinoma studies (I^2 ^= 41.9%). All the studies in our dataset had analyzed only non-small cell lung carcinomas, which include both squamous cell carcinomas and adenocarcinomas. Patients with pulmonary squamous cell carcinoma have a higher mortality rate than those with adenocarcinomas, which might be attributable to confounding factors, such as smoking status and age-related co-morbidities [78,79]. Adenocarcinomas include different subtypes such as acinar, papillary, bronchioalveolar carcinoma and adenocarcinomas with mixed subtypes, which could account for the histological heterogeneity. One study reported that fascin-1 immuno-staining was more common in adenocarcinomas with prevalent invasive components of the acinar, papillary and solid types compared to adenocarcinomas with a prevalent bronchiolo-alveolar component [50]. Thus, the observed heterogeneity in these studies could be due to differences in fascin-1 immunostaining among different subtypes of adenocarcinomas.

Differences in patient selection and disease etiology could also explain the heterogeneity between studies of colorectal carcinomas. Most analyses included all stages of colorectal carcinomas in their specimen sets [6,11,48,54,61-63], nevertheless, multiple studies reported important correlations of fascin-1 expression with increased mortality for stage III and IV tumors [6,24,51,52], as confirmed by our sensitivity analysis results (Table 2B). Historically, the majority of colon adenocarcinomas originate in the left (distal) colon, yet the prevalence of right-sided (proximal) tumors is increasing [80] and multiple studies correlated high fascin-1 expression with right-sided (proximal) colonic tumors [6,24,61,62]. In addition, rectal carcinomas have different biological, clinicopathologic and therapeutic implications from colon carcinomas [52,81]. It would be preferable for future studies to analyze the association of fascin-1 with colorectal carcinomas specifically within stages and according to tumor location [24,52].

For our meta-analysis of the association of fascin-1 with mortality, we extracted multivariable analysis results from most studies, except one, [59], which presented univariable analysis results and seven studies, [19-21,50,53,57,60], which presented results as univariable Kaplan-Meier curves. Subgroup analysis of studies that presented multivariable analysis results showed that fascin-1 protein was associated with a 44% increased risk of mortality. Ideally, meta-analysis would be based on the data of individual patients to allow for standardized control for confounding across all studies [82]. As the hazard ratios from different studies have been adjusted for different confounders, we were unable to assess the independent role of fascin-1 as a new marker over existing markers [68].

## Conclusions

Our meta-analysis study demonstrates that fascin-1 is associated with increased risk of mortality in breast, colorectal and esophageal carcinomas and with increased risk of metastasis in colorectal and gastric carcinomas. The results were stable to a variety of sensitivity analyses and did not vary by predefined subgroups. Due to limitations of the individual studies to date (issues of methodological quality due to retrospective study designs, inadequate sample size or power justification, possible biases due to selective reporting and heterogeneity in study methodologies), adequately powered prospective studies, particularly in breast, colorectal, gastric and esophageal carcinomas, will be needed to fully determine the relative independent prognostic impact of fascin-1. Pooled analysis of all carcinomas within our dataset provides strong evidence that fascin-1 may have potential as a novel biomarker for early identification of aggressive and metastatic tumors. These data will assist rational decision making for focusing on-going efforts investigating fascin-1 as a biomarker onto the most relevant carcinomas.

## Abbreviations

CI: Confidence Interval; HR: Hazard Ratio; I-V: Inverse of the variance; kDa: kiloDalton; MeSH: Medical Subject Headings; mRNA: messenger RNA; PRISMA: Preferred Reporting Items for Systematic Reviews and Meta-Analyses; RR: Risk Ratio; τ^2^: tau-squared.

## Competing interests

The authors declare that they have no conflicts of interest.

## Authors' contributions

RMM and JCA had the idea for and designed the study. VYT searched for and collected the data. RMM and VYT carried out independent review of eligible articles. VYT extracted the data, which was checked by RMM and SJL. RMM and VYT carried out analysis and interpretation of data, with input from JCA. RMM, SJL and VYT carried out secondary data analysis to test the robustness of the data. VYT prepared the first draft. All authors were involved in writing the manuscript and approved the final version.

## Pre-publication history

The pre-publication history for this paper can be accessed here:

http://www.biomedcentral.com/1741-7015/11/52/prepub

## Supplementary Material

Additional file 1 **Supplementary data on the data extraction and analysis methods**. 1. Search terms used. 2. Newcastle-Ottawa Scale results for studies analysing mortality. 3. Alternative scoring methodologies used to assess immunohistochemical staining of Fascin-1. 4. Method used to derive hazard ratios from the Kaplan-Meier curve analysis. Click here for file

Additional file 2 **Study characteristics and key results of papers included in our meta-analysis**. Data were extracted from the indicated publications as described in the Methods. Blank category boxes indicate that this data category was absent from the publication. Click here for file

Additional file 3 **Funnel plot analysis for lymph node metastasis after excluding studies **[8,64]. Black dots represent each study's effect estimate (drawn on a log scale) plotted against its standard error. The outer dashed lines represent the 95% confidence limits around the summary effect estimate, within which 95% of studies are expected to lie in the absence of both biases and heterogeneity. *P*-values are for the results of Egger's test to assess publication bias. Click here for file
