# Supplementary material for: Association of fascin-1 with mortality, disease progression and metastasis in carcinomas: a systematic review and meta-analysis
Source: BMC Med. 2013 Feb 26;11:52. doi: 10.1186/1741-7015-11-52 (PMC3635876; doi:10.1186/1741-7015-11-52)
Supplement: Additional file 1 — Supplementary data on the data extraction and analysis methods. 1. Search terms used. 2. Newcastle-Ottawa Scale results for studies analysing mortality. 3. Alternative scoring methodologies used to assess immunohistochemical staining of Fascin-1. 4. Method used to derive hazard ratios from the Kaplan-Meier curve analysis. [file 1741-7015-11-52-S1.DOCX]

**Additional File 1:**

1. **Search terms used:**
   1. **Fascin-1**

- Fascin/Fascin(s)/Fascin(1)
- MICROFILAMENT PROTEIN(S)
- Actin-bundling protein(s)
- Actin-binding protein(s)
- FSCN(1)
- Fascin homology(1)
  1. **Breast Cancer**
- Breast Cancer
- BREAST NEOPLASM(S)
- CARCINOMA,INTRADUCTAL,NONINFILTRATING
- CARCINOMA,DUCTAL,BREAST
- Breast tumor(s)
- Breast carcinoma(s)/carcinoma(s) of the breast
- Breast adenoma(s)
  1. **Colorectal Cancer**
- COLORECTAL NEOPLASM(s)
- Colorectal cancer(s)
- Colorectal carcinoma(s)
- Colorectal tumour(s)
- COLONIC NEOPLASM(s)
- Colon neoplasm(s)
- Colon tumour(s)
- Colon cancer(s)
- RECTAL NEOPLASM(s)
- Rectal cancer(s)
- Rectal tumour(s)
- Rectum cancer(s)
- Rectum tumour(s)
  1. **Gastric Cancer**
- STOMACH NEOPLASM(S)
- Gastric neoplasm(s)
- Stomach cancer(s)
- Gastric cancer(s)
- stomach carcinoma(s)
- Gastric carcinoma(s)
- Stomach tumo(u)r(s)
- Gastric tumo(u)r(s)
  1. **Lung Cancer**
- LUNG NEOPLASM(S)
- Lung tumour(s)
- Lung Cancer(s)
- Pulmonary neoplasm(s)
- Pulmonary cancer(s)
- NON SMALL CELL LUNG CARCINOMA(S)
- NSCLC
- Non small cell lung cancer(s)
- SMALL CELL LUNG CARCINOMA(S)
- small cell lung cancer(s)
- SCLC
- Oat cell carcinoma(s) of Lung
- BRONCHIAL NEOPLASM(S)
- Bronchi/Bronchial tumour(s)
- Bronchi/Bronchial Cancer(s)
- Bronchial carcinoid(s)
- BRONCHOGENIC CARCINOMA(S)
- Bronchial carcinoma
- BRONCHIOLO-ALVEOLAR ADENOCARCINOMA(S)
- Bronchiolo-alveolar carcinoma(s)
- Bronchiolar carcinoma(s)
- Alveolar adenocarcinoma(s)
- Alveolar carcinoma(s)
  1. **Oesophageal Cancer**
- Esophageal Neoplasm(s)
- Oseophageal Neoplasm(s)
- Esophagus Neoplasm(s)
- Oseophagus Neoplasm(s)
- Esophageal cancer(s)
- Oesophageal cancer(s)
- Esophagus cancer(s)
- Oesophagus cancer(s)
- Esophageal carcinoma(s)
- Oseophageal carcinoma(s)
- Esophagus carcinoma(s)
- Oseophagus carcinoma(s)

| 1. **Newcastle-Ottawa Scale results for studies analysing mortality** | | | |
| --- | --- | --- | --- |
| **Included Studies** | **Selection** | **Comparability** | **Outcome** |
| **Yoder et al [1]** | ** | Not reported | ** |
| **Rodriguez-Pinilla et al [2]** | ** | Not reported | Not reported |
| **Al-Alwan et al [3]** | ** | Not reported | * |
| **Hashimoto et al [4]** | ** | ** | ** |
| **Chan et al [5]** | **** | ** | ** |
| **Jung et al [6]** | ** | * | * |
| **Puppa et al [7]** | **** | ** | ** |
| **Ozerhan et al [8]** | ** | Not reported | Not reported |
| **Oh et al [9]** | ** | * | ** |
| **Vignjevic et al [10]** | * | Not reported | Not reported |
| **Kim et al [11]** | * | Not reported | ** |
| **Pang et al [12]** | * | Not reported | * |
| **Hashimoto et al [13]** | ** | ** | ** |
| **Tsai et al [14]** | ** | Not reported | Not reported |
| **Li et al [15]** | ** | ** | ** |
| **Pelosi et al [16]** | ** | ** | * |
| **Roh et al [17]** | ** | Not reported | ** |
| **Choi et al [18]** | * | Not reported | ** |
| **Zhao et al [19]** | * | Not reported | * |
| **Hashimoto et al [20]** | ** | ** | ** |
| **Hsu et al [21]** | ** | Not reported | * |
| **Zhao et al [22]** | ** | ** | ** |
| **Takikita et al [23]** | ** | ** | ** |
| **Qin et al [24]** | ** | Not reported | ** |
| **Zhang et al [25]** | * | Not reported | ** |
| **Xue et al [26]** | * | Not reported | * |

Notes: The studies are judged on three broad perspectives using a nine point scale according to the Newcastle-Ottawa quality assessment scale: selection of the study group (0-4 points), comparability of cohorts (0-2 points) and ascertainment of outcome (0-3 points). *=1 point

**3) Alternative scoring methodologies used to assess immunohistochemical staining of fascin-1:**

- 1. Takikita et al [23] and Pang et al [12] categorised a score of ≤3 as negative fascin-1 expression by the tumour. Because an overall score of 3 could possibly include fascin-1-positive cells, for these two studies, we categorised a score of ≤3 as low fascin-1 expression and a score of ≥4 as high fascin-1 expression.
  2. For papers [8,16-19] that presented their data as negative, low, or high scores, we dichotomised the scoring of fascin-1 as negative versus positive (ie, low and high were combined). For Jung et al [6], we dichotomised fascin-1 as high versus low, because the authors had categorised negative staining as the scores of 0-2 which could include fascin-1-positive tumour cells.

1. **Method used to derive hazard ratios from the Kaplan-Meier curve analysis:**

We extracted data from the Kaplan-Meier curve using the method of Tierney et al [27]. For their method, the Kaplan-Meier curve was divided into a number of time intervals and a number of iterative calculations were carried out to estimate the number of events during each interval and the number of patients who were: i), event-free at the start of the interval; ii), censored during the interval, and iii), at risk during the interval. HRs and their variance were then calculated for each time interval. The HR and variance for the whole curve were derived by combining the estimates across the time intervals.

We extracted data from the Kaplan-Meier curve analysis of the study by Tsai et al [14] by using the method of Tierney et al [27]. The full sample set included 100 patients. The Kaplan-Meier curve included 75 patients and we did not have data on the proportion of fascin-1 positive or negative tumour specimens in these 75 patients. We estimated the log hazard ratio from the study by assuming that the proportion of fascin-1-positive versus -negative tumour specimens was the same in the 75 patients included in the survival analysis as in the full sample set from 100 patients.

**References for Additional File 2**

1. Yoder BJ, Tso E, Skacel M, Pettay J, Tarr S, Budd T, Tubbs RR, Adams JC, Hicks DG: **The expression of fascin, an actin-bundling motility protein, correlates with hormone receptor-negative breast cancer and a more aggressive clinical course**. *Clinical Cancer Research* 2005, **11**:186-192.

2. Rodriguez-Pinilla SM, Sarrio D, Honrado E, Hardisson D, Calero F, Benitez J, Palacios J: **Prognostic significance of basal-like phenotype and fascin expression in node-negative invasive breast carcinomas**. *Clinical Cancer Research* 2006, **12**:1533-1539.

3. Al-Alwan M, Olabi S, Ghebeh H, Barhoush E, Tulbah A, Al-Tweigeri T, Ajarim D, Adra C: **Fascin Is a Key Regulator of Breast Cancer Invasion That Acts via the Modification of Metastasis-Associated Molecules**. *PLoS ONE [Electronic Resource]* 2011, **6**.

4. Hashimoto Y, Skacel M, Lavery IC, Mukherjee AL, Casey G, Adams JC: **Prognostic significance of fascin expression in advanced colorectal cancer: an immunohistochemical study of colorectal adenomas and adenocarcinomas**. *BMC Cancer* 2006, **6**.

5. Chan C, Jankova L, Fung CLS, Clarke C, Robertson G, Chapuis PH, Bokey L, Lin BPC, Dent OF, Clarke S: **Fascin Expression Predicts Survival After Potentially Curative Resection of Node-positive Colon Cancer**. *American Journal of Surgical Pathology* 2010, **34**:656-666.

6. Jung EJ, Lee JH, Min BW, Kim YS, Choi JS: **Clinicopathologic significance of fascin, extracellular matrix metalloproteinase inducer, and ezrin expressions in colorectal adenocarcinoma**. *Indian Journal of Pathology and Microbiology* 2011, **54**:32-36.

7. Puppa G, Maisonneuve P, Sonzogni A, Masullo M, Chiappa A, Valerio M, Zampino MG, Franceschetti I, Capelli P, Chilosi M, Menestrina F, Viale G, Pelosi G: **Independent prognostic value of fascin immunoreactivity in stage III-IV colonic adenocarcinoma**. *British Journal of Cancer* 2007, **96**:1118-1126.

8. Ozerhan IH, Ersoz N, Onguru O, Ozturk M, Kurt B, Cetiner S: **Fascin expression in colorectal carcinomas**. *Clinics* 2010, **65**:157-164.

9. Oh SY, Kim YB, Suh KW, Paek OJ, Moon HY: **Prognostic impact of fascin-1 expression is more significant in advanced colorectal cancer**. *J Surg Res* 2012, **172**:102-108.

10. Vignjevic D, Schoumacher M, Gavert N, Janssen KP, Jih G, Lae M, Louvard D, Ben-Ze'ev A, Robine S: **Fascin, a novel target of beta-Catenin-TCF signaling, is expressed at the invasive front of human colon cancer**. *Cancer Research* 2007, **67**:6844-6853.

11. Kim BG, Ha KW, Park JS, Yoo JH: **Immunohistochemical studies of fascin, MMP-9 overexpression, and proliferating index as prognostic factors in cases of a colon adenocarcinoma. [Korean]**. *Journal of the Korean Society of Coloproctology* 2009, **25**:393-400.

12. Pang Q, Zhang X, Zhang J, Li Y, Wu W, Wang H, Cui A, Zhang Y: **Correlative studies on expression of CXCR4, CXCR12, Fascin and E-cadherin in colorectal carcinoma with lymphatic metastasis. [Chinese]**. *Chinese Journal of Clinical Oncology* 2009, **36**:926-929+933.

13. Hashimoto Y, Shimada Y, Kawamura J, Yamasaki S, Imamura M: **The prognostic relevance of fascin expression in human gastric carcinoma**. *Oncology* 2004, **67**:262-270.

14. Tsai WC, Jin JS, Chang WK, Chan DC, Yeh MK, Cherng SC, Lin LF, Sheu LF, Chao YC: **Association of cortactin and fascin-1 expression in gastric adenocarcinoma: Correlation with clinicopathological parameters**. *Journal of Histochemistry and Cytochemistry* 2007, **55** :955-962.

15. Li X, Zheng H, Hara T, Takahashi H, Masuda S, Wang Z, Yang X, Guan Y, Takano Y: **Aberrant expression of cortactin and fascin are effective markers for pathogenesis, invasion, metastasis and prognosis of gastric carcinomas**. *International Journal of Oncology* 2008, **33** :69-79.

16. Pelosi G, Pastorino U, Pasini F, Maissoneuve P, Fraggetta F, Iannucci A, Sonzogni

A, De Manzoni G, Terzi A, Durante E, Bresaola E, Pezzella F, Viale G:

**Independent prognostic value of fascin immunoreactivity in stage I nonsmall**

**cell lung cancer**. *British Journal of Cancer* 2003, **88**:537 547.

17. Roh MS, Um SJ, Choi Y, Kim KN, Pil JC, Lee SK, Son C, Yang D: **Prognostic significance of fascin expression in stage I non-small cell lung cancer. [Korean]**. *Tuberculosis and Respiratory Diseases* 2008, **65**:105-109.

18. Choi PJ, Yang DK, Son CH, Lee KE, Lee JI, Roh MS: **Fascin immunoreactivity for preoperatively predicting lymph node metastases in peripheral adenocarcinoma of the lung 3 cm or less in diameter**. *Eur J Cardiothorac Surg* 2006, **30**:538-542.

19. Zhao J, Zhou Y, Zhang Z, Tian F, Ma N, Liu T, Gu Z, Wang Y: **Upregulated fascin1 in non-small cell lung cancer promotes the migration and invasiveness, but not proliferation**. *Cancer Lett* 2010, **290**:238-247.

20. Hashimoto Y, Ito T, Inoue H, Okumura T, Tanaka E, Tsunoda S, Higashiyama M, Watanabe G, Imamura M, Shimada Y: **Prognostic significance of fascin overexpression in human esophageal squamous cell carcinoma**. *Clinical Cancer Research* 2005, **11**:2597-2605.

21. Hsu KF, Lin CK, Yu CP, Tzao C, Lee SC, Lee YY, Tsai WC, Jin JS: **Cortactin, fascin, and survivin expression associated with clinicopathological parameters in esophageal squamous cell carcinoma**. *Diseases of the Esophagus* 2009, **22**:402-408.

22. Zhao Q, Shen JH, Shen ZY, Wu ZY, Xu XE, Xie JJ, Wu JY, Huang Q, Lu XF, Li EM*,* Xu LY: **Phosphorylation of fascin decreases the risk of poor survival in patients with esophageal squamous cell carcinoma**. *Journal of Histochemistry and* *Cytochemistry* 2010, **58**:979-988.

23. Takikita M, Hu N, Shou JZ, Giffen C, Wang QH, Wang C, Hewitt SM, Taylor PR: **Fascin and CK4 as biomarkers for esophageal squamous cell carcinoma**. *Anticancer Research* 2011, **31**:945-952.

24. Qin YR, Tang H, Qiao JJ, Li FF, Ai JY: **[Expression of fascin in human esophageal squamous cell carcinoma and its clinical significance]**. *Nan Fang Yi Ke Da Xue Xue Bao* 2011, **31**:1216-1219.

25. Zhang H, Xu L, Xiao D, Xie J, Zeng H, Cai W, Niu Y, Yang Z, Shen Z, Li E: **Fascin is a potential biomarker for early-stage oesophageal squamous cell carcinoma**. *Journal of Clinical Pathology* 2006, **59**:958-964.

26. Xue LY, Song YM, Tong T, Luo W, Dong LJ, Zou SM, Zheng S, Bi R, Zhan QM, Lu N: **Expression of fascin and cytokeratin 14 in esophageal squamous cell carcinoma. [Chinese]**. *National Medical Journal of China* 2007, **87**:2494-2498.

27. Tierney JF, Stewart LA, Ghersi D, Burdett S, Sydes MR: **Practical methods for incorporating summary time-to-event data into meta-analysis**. *Trials* 2007, **8**:16.
