# Supplementary material for: Association of fascin-1 with mortality, disease progression and metastasis in carcinomas: a systematic review and meta-analysis
Source: BMC Med. 2013 Feb 26;11:52. doi: 10.1186/1741-7015-11-52 (PMC3635876; doi:10.1186/1741-7015-11-52)
Supplement: Additional file 2 — Study characteristics and key results of papers included in our meta-analysis. Data were extracted from the indicated publications as described in the Methods. Blank category boxes indicate that this data category was absent from the publication. [file 1741-7015-11-52-S2.DOCX]

| **Additional file 2** Study characteristics and key results | | | | | | | | | | | | | | |
| --- | --- | --- | --- | --- | --- | --- | --- | --- | --- | --- | --- | --- | --- | --- |
| **Author** | **Country** | **Carcinoma type** | **Mean/ median (range) age** | **Mean/ median (range)**  **follow-up** | **Categorisation and no. of cases** | **T** | **N** | **M** | **Stage** | **Histology** | **Type of Section** | **Antibody used**  **(Dilution)** | **Mortality analysis** | **Adjustments** |
| **Breast Carcinoma** | | | | | | | | | | | | | | |
| Yoder  et al [19] | USA | Primary invasive breast carcinoma | <50yrs: n=66  >50yrs: n=144 | 67  (1-106) mths | -ve: <10% (n=177); +ve: >10%  (n=33) |  |  |  |  | Bloom-Richardson grade 3:  n=29 (19) | TMA | Dako (Clone 55K2) (1:50) | KM curve |  |
| Rodriguez-Pinilla et al [20] | Spain | Familial and sporadic node-negative breast carcinomas | 53 (27-87 ) yrs | 101 (4-185) mths | -ve: <10% (n=153); +ve: >10% (n=55) | pT1:  n=117 (25)  pT2:  n=70 (21) |  |  |  | G1:  n=54 (7)  G2:  n= 57 (11)  G3:  n= 84 (32) | TMA | Dako (Clone 55K2) (1:50) | KM curve |  |
| Al-Alwan et al [21] | Saudi Arabia | Invasive ductal carcinoma | <40yrs: n=21  ≥40yrs: n=50 |  | -ve: <5% (n=49)  +ve: >5% (n=22) |  |  |  |  | G 1:  n=2 (1)  G2:  n=33 (6)  G 3:  n=36 (15) | Tissue Sections | Dako (Clone 55K2) (1:200) | KM curve |  |
| **Colorectal Carcinoma** | | | | | | | | | | | | | | |
| Hashimoto et al [6] | USA | Colorectal adenocarcinoma  (All stages) | 64.5 (32- 95) yrs | 38 mths | Low: 0 or 1 (n=113);  High: 2 or 3 (n=18) |  | -ve: n=78(10)  +ve:  n=53(8) |  | TNM staging:  Stage I/II: n= 69(9)  Stage III/IV:  n=62 (9) |  | TMA | Dako (Clone 55K2) (1:50) | Multi-variable | age>65, gender, LN and distant Mets, tumour location |
| Puppa et al [52] | Italy | Colonic adenocarcinomas  (Stage III/IV) | Male:  66 (38-84)yrs  Female: 65 (30-84) yrs | Censored at 6yrs | -ve:  (n=66);  +ve: 1-100/ >100 (n=162) | T1:  n=2 (2)  T2:  n=12 (9)  T3:  n=185 (129)  T4:  n=29 (22) | N1:  n=139 (97)  N2:  n=89 (65) | M0:  n=170 (115)  M1:  n=58 (47) |  | G1:  n=16 (11)  G2:144 (93)  G3:68 (58) | Tissue Sections | Dako (Clone 55K2) | Multi-variable | age, sex, histology and stage |
| **Author** | **Country** | **Carcinoma type** | **Mean/ median (range) age** | **Mean/ median (range)**  **follow-up** | **Categorisation and no. of cases** | **T** | **N** | **M** | **Stage** | **Histology** | **Type of Section** | **Antibody used**  **(Dilution)** | **Mortality analysis** | **Adjustments** |
| **Colorectal Carcinoma (Continued)** | | | | | | | | | | | | | | |
| Vignjevic et al [11] | France | Colon carcinoma |  |  | -ve: (n=44);  +ve:(n=35) | T2:  n=33(11)  T3:  n=36 (19)  T4:  n=10 (5) | N0:  n=43 (12)  N1, N2, N3:  n=36 (23) | M0:  n=58 (23)  M1:  n=21 (14) | TNM staging:  Stage 1:  n=21 (5)  Stage II:  n=23 (7)  Stage III: n= 17 (12)  Stage IV:  n=21 (15) | G1:  n=45 (17)  G2:  n=82 (44)  G3:  n=17 (11)  G4:  n= 4 (4) | TMA | Dako (Clone 55K2) |  |  |
| Kim et al [62] | Korea | Colon adenocarcinoma  (All stages) | ≤65 yrs: (n=56);  >65 yrs: (n=64) |  | -ve: <10%  (n = 67);  +ve: >10% (n=53) | T1/2:  n=10 (3)  T3/4:  n=110 (50) | -ve :  n=50 (15)  +ve:  n=70 (38) |  |  | G1/G2 :  n=109 (45)  G3 :  n=11 (9) | Tissue sections | Dako (Clone 55K2) (1:50) |  |  |
| Ozerhan et al [61] | Turkey | Colorectal adenocarcinoma  (Stage III/IV for disease-specific mortality) | 64 (21-88) yrs | 28.29 (1- 67) mths | -ve: 0 (n=108);  +ve: ≤10/ >10* (n=59) | T1/T2 :  n=17 (5)  T3 /T4 :  n=150 (54) | N0 :  n=92 (24)  N1 :  n=40 (17)  N2 :  n=35 (18) | M0 :  n=137 (46)  M1 :  n=30 (13) | TNM staging:  Stage I/II:  n=88 (24)  Stage III/IV:  n=79 (35) | G1:  n=6 (4)  G2:  n=129 (42)  G3:  n=19 (7) | Tissue sections | Neomarkers (1 :80)  (same as Labvision) | 2 yr mortality rates |  |
| Chan et al [24] | Australia | Colon adenocarcinoma  (Stage C) | <75 yrs:  n=312;  >75 yrs:  n=158 | 341 patients (discharged alive but died subsequently)  Median follow-up (range):  35 (0.3-351 mths)  103 patients (surviving), median (range):  125 (73-303 mths) | Low (<40%): n=308  High (>40%): n=138 | T1/T2:  n=27 (1)  T3/T4 :  n=419 (137) |  |  |  | G1/G2:  n=276 (60)  G3:  n=170 (78) | TMA | Dako (Clone 55K2) (1:100) | Multivariable | Age >75, high grade, venous invasion, apical node involvement, free serosal surface involvement, T4, no adjuvant chemotherapy |
| **Author** | **Country** | **Carcinoma type** | **Mean/ median (range) age** | **Mean/ median (range)**  **follow-up** | **Categorisation and no. of cases** | **T** | **N** | **M** | **Stage** | **Histology** | **Type of Section** | **Antibody used**  **(Dilution)** | **Mortality analysis** | **Adjustments** |
| **Colorectal Carcinoma (Continued)** | | | | | | | | | | | | | | |
| Pang et al [63] | China | Colorectal Carcinoma  (All stages) | 67 (18-83) yrs |  | Low: <3  (n=66);  High: ≥3 * (n=60) |  | -ve:  n=69 (27)  +ve:  n=57 (33) |  | Duke’s staging:  A+B :  n=32 (10)  C+D:  n=94 (50) | G1:  n=34 (12)  G2:  n= 46 (17)  G3:  n=46 (31) | Tissue sections | Neomarkers  (same as Labvision) |  |  |
| Oh et al [51] | Korea | Colorectal carcinoma  (Stage III) | 59.2 (19-80) yrs | 42.8 (3-84 ) mths | -ve: <10% (n=52); +ve: >10% (n=74) | T2 :  n=10 (8)  T3 :  n=111 (63)  T4 :  N=5(3) | N1:  n=85 (53)  N2:  n=41 (21) |  | TNM staging:  Stage IIIA:  n=7 (6)  Stage IIIB:  n=78 (47)  Stage IIIC:  n=41 (21) | G1:  n=8 (6)  G2:  n=89 (48)  G3:  n=29 (20) | TMA | Dako (Clone 55K2) (1:50) | Multi-variable (a) | For mortality: Nodal Status, TNM stage and venous invasion #  For distant mets: adjusted for nodal status, and tumour location |
| Jung et al [54] | Korea | Colorectal adenocarcinoma  (All Stages) | 58 (16-83) yrs |  | Low: 0-5  (n=159)  High: 6-9 * (n=51) | T1/T2:  n=24 (1)  T3/T4: n=186 (50) | N0: n=119 (26)  N1:  n=91 (25) | M0:  n=198 (47)  M1:  n=12 (4) | TNM staging:  Stage I/II: n=117 (25)  Stage III/IV: n=93 (26) |  | TMA | Thermo Scientific  (1:100) | Multi-variable (a) | LN and distant Mets, tumour depth, ezrin and EMMPRIN  # |
| **Gastric Carcinoma** | | | | | | | | | | | | | | |
| Hashimoto et al [55] | Japan | Gastric adenocarcinoma | 63 (31–87) yrs | 68.5 (1-121) mths | -ve: <5% (n=160);  +ve:>5%  (n=54) | Tis-T2:  n=178 (38)  T3-T4:  n=37 (16) | -ve:  n=118 (20)  +ve:  n=96 (34) | M0:  N=175 (39)  M1:  n=39 (15) | TNM Stage:  Stage 0/I/II:  n=140 (25)  Stage III/IV:  n=74 (29) | G1:  n=43 (6)  G2:  n=74 (9)  G3:  n=89 (19) | Tissue sections | Dako (Clone 55K2) (1:50) | Multi-variable | Gender, age, serosal invasion, nodal status, Mets, and R classification |
| Tsai et al [57] | Taiwan | Gastric adenocarcinoma | 66.7 (33-89) yrs | 3 years | -ve: <5%  (n=23);  +ve:>5%  (n=77) | T1:  n=14 (8)  T2:  n=14 (9)  T3:  n=49 (39)  T4:  n=23 (21) | N0:  n=36 (24)  N1:  n=28 (21)  N2:  n=20 (16)  N3:  n=16 (16) | M0:  n=88 (66)  M1:  n=12 (11) | AJCC Stage:  Stage 1:  n=25 (16)  Stage 2:  n=15 (13)  Stage 3:  n=39 (30)  Stage 4:  n=21 (18) | G1:  n=20 (13)  G2:  n=20 (14)  G3:  n=60 (50) | TMA | Neomarkers (1:100)  (same as Labvision) | KM curve |  |
| **Author** | **Country** | **Carcinoma type** | **Mean/ median (range) age** | **Mean/ median (range)**  **follow-up** | **Categorisation and no. of cases** | **T** | **N** | **M** | **Stage** | **Histology** | **Type of Section** | **Antibody used**  **(Dilution)** | **Mortality analysis** | **Adjustments** |
| **Gastric Carcinoma (Continued)** | | | | | | | | | | | | | | |
| Li et al [56] | China  Japan | Gastric carcinoma | 65.9 (29-91) yrs | 35.9 (0.2-146.4) mths | -ve: <5% (n=378);  +ve:>5%  (n=131) | Tis-T1:  n=275 (55)  T2-T4:  n=234 (76) | -ve:  n=329 (75)  +ve:  n=180 (56) |  | UICC Staging  Stage 0-1:  n=303 (63)  Stage II-IV:  n=206 (68) |  | TMA | Labvision (FCN01) | Multi-variable |  |
| **Lung Carcinoma** | | | | | | | | | | | | | | |
| Pelosi et al [50] | Italy | Non-small cell lung cancer (NSCLC)  (Stage I) | Male:  63 (35-80) yrs  Female: 62 (47-76) yrs | 80 (2–159 mths) | -ve: <5%  (n=23);  +ve:>5% * (n=189) |  |  |  |  |  | Tissue sections | Dako (Clone 55K2) | KM curve |  |
| Choi et al [64] | Korea | Bronchioloalveolar carcinomas  (BAC), adenocarcinomas with BAC components and invasive  carcinomas (mixed), and invasive adenocarcinomas without  BAC components (invasive) | 58 (40-76 yrs) |  | -ve: <5%  (n=19);  +ve:>5% * (n=30) |  | -ve:  n=33 (15)  +ve:  n=16 (15) |  | Pathalogic Stage:  Stage IA:  n=33 (15)  Stage IIA:  n=6 (5)  Stage IIIA:  n=10 (6) |  | TMA | Dako (Clone 55K2) (1:50) |  |  |
| Roh et al [53] | Korea | Non-small cell lung cancer (NSCLC) |  |  | -ve: <5%  (n=22);  +ve:>5% * (n=59) |  |  |  | TNM stage Stage IA:  n=38 (25)  Stage IB:  n=43 (34) | G1:  n= 26 (20)  G2:  n=35 (27)  G3:  n=8 (6) | Tissue sections | Dako (Clone 55K2) (1:50) | KM curve |  |
| Zhao et al [65] | China | Non-small cell lung cancer (NSCLC) | <60 yrs: (n=50);  ≥60yrs: (n=48) |  | -ve: 0  (n=16);  +ve: 1+ to 3+ (n=82) |  | N0:  n=29 (20)  N1:  n=40 (35)  N2:  n=29 (27) |  | TNM Stage:  Stage I:  n=14 (7)  Stage II:  n=39 (32)  Stage III:  n=45 (43) |  | Tissue sections | Abcam (1:300) |  |  |
| **Author** | **Country** | **Carcinoma type** | **Mean/ median (range) age** | **Mean/ median (range)**  **follow-up** | **Categorisation and no. of cases** | **T** | **N** | **M** | **Stage** | **Histology** | **Type of Section** | **Antibody used**  **(Dilution)** | **Mortality analysis** | **Adjustments** |
| **Oesophageal Carcinoma** | | | | | | | | | | | | | | |
| Hashimoto et al [9] | Japan | Oesophageal squamous cell carcinoma (ESCC) | 65 (43- 90) yrs | 40.1 mths  (2 to 146 mths) | Immuno-reactivity:  Low: <75% (n=55)  High: >75% (n=145) |  | -ve:  n=73 (44)  +ve:  n=127 (101) | M0:  n=167 (118)  M1:  n=33 (27) | TNM Stage:  Stage I/II:  n=103 (68)  Stage III/IV:  n=97 (77) | G1:  n=42 (26)  G2:  n=107 (83)  G3:  n=51 (36) | Tissue sections | Dako (Clone 55K2) (1:50) | Multi-variable | Gender, age, extent of tumour, nodal status and Mets. |
| Zhang et al [8] | China | Oesophageal squamous cell carcinoma (ESCC) | 57 (40-75) yrs |  | -ve: <5%  (n=22);  +ve:>5%  (n=40) | T1/T2:  n= 5(4)  T3/T4:  n=57 (36) | N0:  n=37 (20)  N1:  n=25 (20) | M0:  n= 60 (39)  M1:  n=2 (1) | Stage I/IIA/IIB):  n=38 (21)  Stage III/IV:  n=24 (19) | G1:  n=21 (12)  G2:  n=21 (13)  G3:  n=20 (15) | Tissue sections | Dako (Clone 55K2) (1:800) |  |  |
| Xue et al [66] | China | Oesophageal squamous cell carcinoma (ESCC) | 59.5 (33- 78) yrs |  | Low: <9  (n=64);  High:≥9  (n=52) |  | N0 #  n=53 (25)  N1  n=63 (27) |  | pTNM stage (according to AJCC/ UICC staging 6th edition) #  Stage IIA/IIB:  n=54 (28)  Stage III/ IV:  n=62 (24) |  | TMA | Dako (Clone 55K2) (1:50) |  |  |
| Hsu et al [59] | Taiwan | Oesophageal squamous cell carcinoma (ESCC) | 61.4 (34-84) yrs | 5 yr follow-up | Low:<245 (n=23);  High:≥245 (n=23) |  | M0:  n=241 (83)  M1:  n= 13 (7) |  | AJCC Stage: **  G1: 169.3 +/- 21.7  G2: 207.0 +/- 27.2  G3: 247.5 +/- 63.5 |  | TMA | Neomarkers (1:100)  (same as Labvision) | Uni-variable |  |
| Zhao et al [58] | China | Oesophageal squamous cell carcinoma (ESCC) | 55 (32-73) yrs | 25.15 mths  (1.7- 131.3) mths) | Low: <9  (n=164);  High:≥9  (n=90) | T1/T2: #  n=36 (12)  T3/T4:  n=218  (78) | N0: #  n=137 (46)  N1:  n=117 (44) | M0: #  n=241 (83)  M1:  n= 13 (7) |  | G1: #  n=54 (17)  G2:  n=162 (64)  G3:  n=38 (9) | TMA | Dako (Clone 55K2) (1:100) | Multi-variable | Gender, age, and factors p<0.1, excluding pTNM stage |
| **Author** | **Country** | **Carcinoma type** | **Mean/ median (range) age** | **Mean/ median (range)**  **follow-up** | **Categorisation and no. of cases** | **T** | **N** | **M** | **Stage** | **Histology** | **Type of Section** | **Antibody used**  **(Dilution)** | **Mortality analysis** | **Adjustments** |
| **Oesophageal Carcinoma (Continued)** | | | | | | | | | | | | | | |
| Takikita  et al  [7] | China | Oesophageal squamous cell carcinoma (ESCC) | 57 yrs |  | Low: 0-3 (n=102);  High: 4-12 * (n=129) |  | -ve: #  n=126 (77)  +ve:  n=104 (52) |  | Stage I: #  n=1 (0)  Stage II:  n=29 (17)  Stage III:  n=198 (112)  Stage IV:  n=2 (0) | G1: #  n=119 (74)  G3:  n=112 (55) | TMA | Dako (Clone 55K2) (1:50) | Multi-variable | Gender, age, tobacco use, alcohol use, family history of Upper GI cancer, grade, stage, Mets and differentiation |
| Qin et al  [60] | China | Oesophageal squamous cell carcinoma (ESCC) | ≤60 yrs  (n=128);  >60yrs: (n=113) |  | -ve: <5%  (n=75);  +ve:>5% ( n=166) |  | -ve:  n=130 (81)  +ve:  n=111 (85) |  | Stage I:  n= 8 (2)  Stage II:  n=148 (98)  Stage III:  n=85 (66) | G1:  n=29 (15)  G2:  n=158 (115)  G3:  n=54 (36) | TMA | Dako (Clone 55K2) | KM curve |  |

Notes: LN=Lymph node; Mets=Metastasis; GI=Gastrointestinal; KM=Kaplan-Meier; mths= months; yrs=years; R classification= Residual tumour; _*_ = Data pooled using alternative categorisation methods; ** = mean +/- standard error; AJCC=American Joint Committee on Cancer; UICC=Union Internationale Contre le Cancer; TNM stage=Tumour Node Metastasis stage; TMA: tissue microarray; G1=Low grade or well-differentiated;G2=Moderate grade or Moderately differentiated; G3=High grade or Poorly differentiated; T=Size or direct extent of primary tumour; N=degree of spread to regional lymph nodes; M=presence of metastasis; #=Data obtained from contacting authors; (a) Definition of mortality provided after contacting authors
