# Supplementary figures and images for: Association of fascin-1 with mortality, disease progression and metastasis in carcinomas: a systematic review and meta-analysis
Source: BMC Med. 2013 Feb 26;11:52. doi: 10.1186/1741-7015-11-52 (PMC3635876; doi:10.1186/1741-7015-11-52)

Funnel Plot for Lymph node metastasis after excluding studies [8,64]

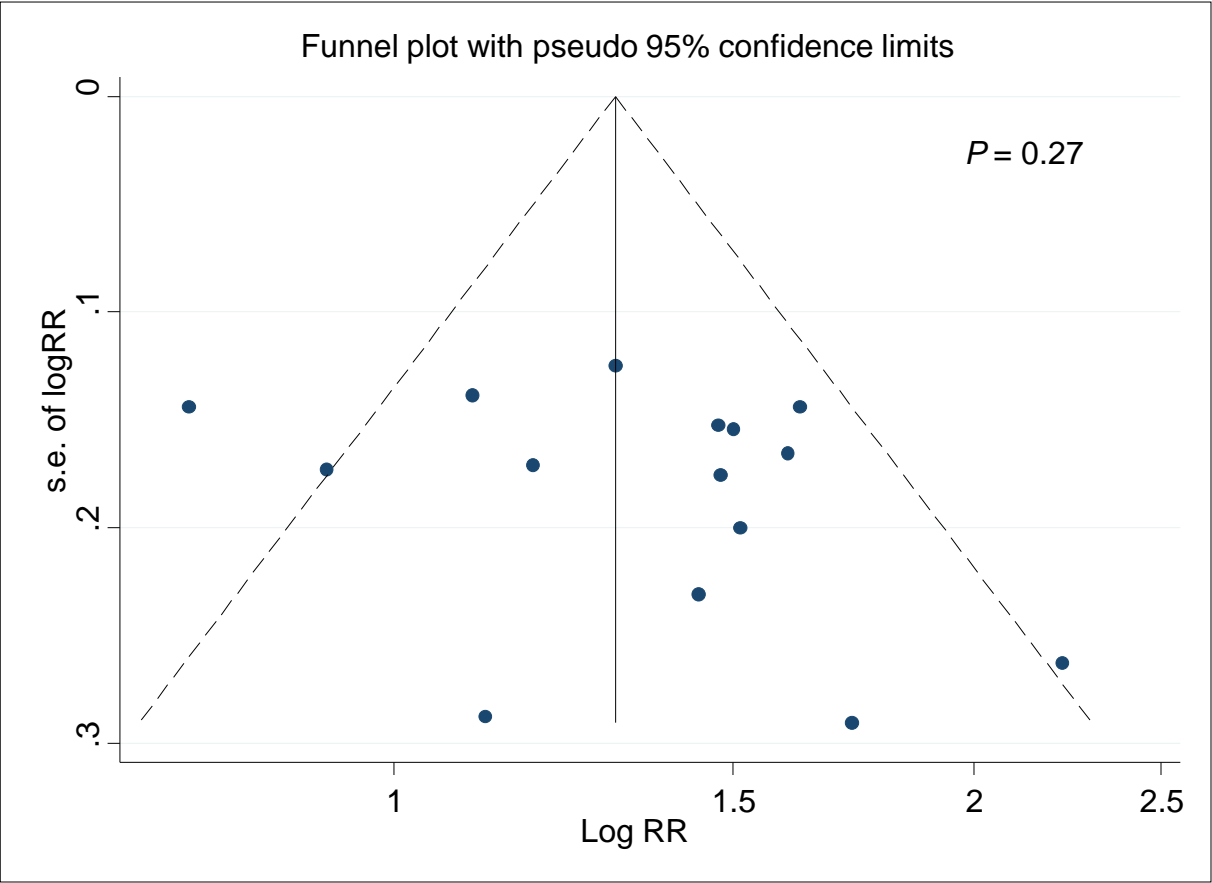

Supplement: Additional file 3 — Funnel plot analysis for lymph node metastasis after excluding studies [8,64]. Black dots represent each study's effect estimate (drawn on a log scale) plotted against its standard error. The outer dashed lines represent the 95% confidence limits around the summary effect estimate, within which 95% of studies are expected to lie in the absence of both biases and heterogeneity. P-values are for the results of Egger's test to assess publication bias. [file 1741-7015-11-52-S3.PDF]
